# Main Problems Using DEM Modeling to Evaluate the Loose Soil Collection by Conceptual Machine as a Background for Future Extraterrestrial Regolith Harvesting DEM Models

**DOI:** 10.3390/mi12111404

**Published:** 2021-11-15

**Authors:** Przemysław Młynarczyk, Damian Brewczyński

**Affiliations:** Faculty of Mechanical Engineering, Cracow University of Technology, 31-155 Kraków, Poland; damian.brewczynski@pk.edu.pl

**Keywords:** DEM, space mining, 3D printing, regolith, conceptual design, product development

## Abstract

Nowadays, rapid product development is a key factor influencing a company’s success. In the Space 4.0. era, an integrated approach with the use of 3D printing and DEM modeling can be particularly effective in the development of technologies related to space mining. Unfortunately, both 3D printing and DEM modeling are not without flaws. This article shows the possibilities and problems resulting from the use of DEM simulation and 3D printing simultaneously in the rapid development of a hypothetical mining machine. For the subsequent development of the regolith harvesting model, loose soil harvesting simulations were performed and the underlying problems were defined and discussed. The results show that it is possible to use both technologies simultaneously to be able to effectively and accurately model the behavior of this type of machine in various gravitational conditions in the future.

## 1. Introduction

Space mining has become a very popular topic in modern engineering. In recent years, mainly thanks to commercial companies, the topic of extraterrestrial travel and related engineering problems has become more and more relevant. Apart from the legal and sociological aspects, the most important problem seems to be the supply of raw materials to the Earth’s orbit and beyond. As it is very expensive to carry sources from earth, scientists, engineers and owners of commercial space companies are now looking towards the moon as a relatively cheap source of raw materials [1,2,3]. In terrestrial conditions, standard development methods of technologies intended to work in a space environment are very costly and time consuming. While the creation of laboratories mapping space conditions in terms of pressure, radiation, or temperature is well known and quite simple, getting rid of the phenomena generated by Earth’s gravity requires great expenditure and the effect is only partial. In the works [4,5], the problems that arose during the design and use of regolith digging equipment were presented. In the paper [6], an overview of various types of concepts that could be used for the extraction of regolith in conditions of reduced gravity were described. Among the many different concepts of regolith excavation mechanisms, the most common type of design is the bucket ladder. The mechanism concept presented in this article is a complete novelty, so DEM modeling of such a machine has not appeared in the literature so far. Unfortunately, apart from a certain amount of knowledge about the structure of extraterrestrial soils on which the probe landings take place, it is still not known how they behave under low-gravity conditions. DEM simulations, which are currently effectively used in terrestrial soil modeling, can be used to model the interaction of soil grains with each other under low-gravity conditions [7,8,9]. By designing and developing a machine that interacts with the lunar or Martian regoliths, the created model can be tested and adjusted by interacting with the regolith in terrestrial conditions on a test bench in an Earth laboratory. The model prepared in this way can be used later for simulations for other gravitational conditions. Three-dimensional printing is already very common in the preparation of prototypes and elements of research test stands [10,11,12]. The use of 3D printing techniques in this approach allows for quick geometry modification and express testing of other configurations and it is also used for space applications [13,14]. Unfortunately, this approach also has many disadvantages, some of which are covered in this article. However, as this kind of research is not expensive, this approach to designing future space mining machines should be developed as much as possible. Various approaches to space mining can be found in the scientific literature [15,16,17], but in the near future, the mechanical extraction of raw material still seems to be the most likely approach. The main aim of this article was to show the problems and to highlight the advantages of DEM modeling of the new concept of a machine as a future regolith harvesting machine concept.

## 2. Investigated Case

**Figure 1:**
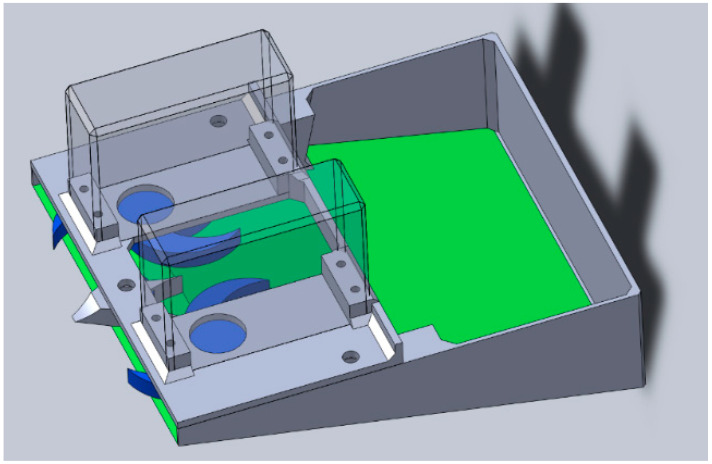
Hypothetical regolith harvesting machine CAD model.

In order to identify the strengths and weaknesses of the coupled “DEM modeling–3D printing” design approach, the authors designed a hypothetical regolith harvesting machine presented in the Figure 1.

**Figure 2:**
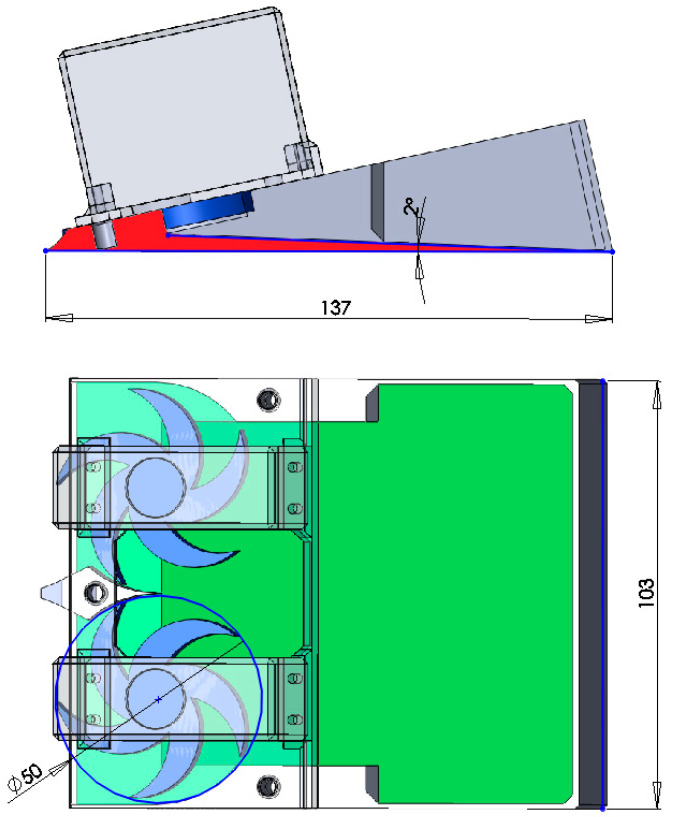
Hypothetical harvester dimensions (in mm).

The main function of the machine is to peel a thin surface layer of soil using two rotating blades and to collect particles via gravity at the back of the machine. In order to reduce the duration of the simulation for relatively small particles (1 mm), a small machine was designed. The exact dimensions of the concept are shown in the Figure 2.

## 3. Simulation Model

Nowadays, when computing resources are growing, the use of the discrete element method (DEM) to calculate the interaction of bulk materials is becoming a common standard. Although DEM calculations are still time consuming and require large computing power, in the near future they will revolutionize the design process in the same way that FEM and CFD simulations have. All simulations presented in this paper were performed using Ansys/Rocky software. The basis for DEM calculations in Rocky software is to follow the path of each particle using two equations—one for particle translation motion (1) and second for particle rotation motion (2) [18,19]:

(1) $$m_{p}\cdot \frac{dv_{p}}{dt}=F_{p}+m_{p}\cdot g$$

(2) $$I_{p}\cdot \frac{d\omega_{p}}{dt}=T_{p}$$

where:

*m_p_*—particle mass;

*v_p_*—particle velocity;

*F_p_*—contact forces on the particle;

*g*—gravitational acceleration;

*I_p_*—particle moment of inertia tensor;

*T_p_*—torque on the particle;

*ω_p_*—particle angular velocity.

**Figure 3:**
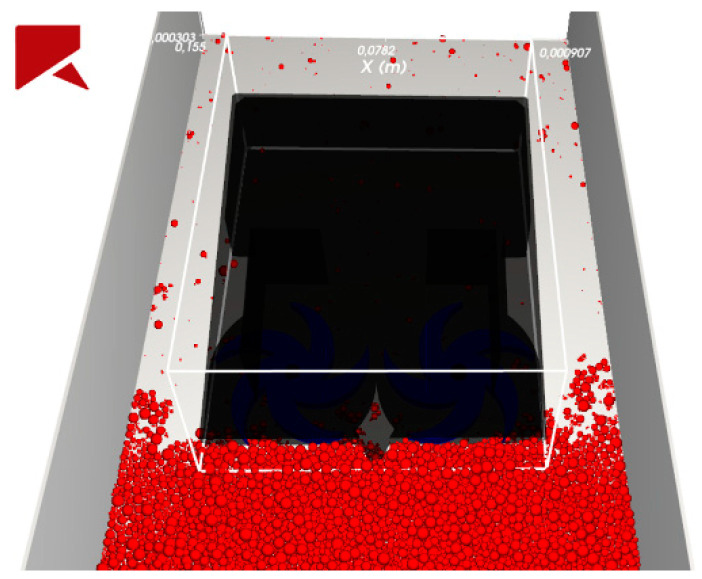
Starting conditions—front view.

**Figure 4:**
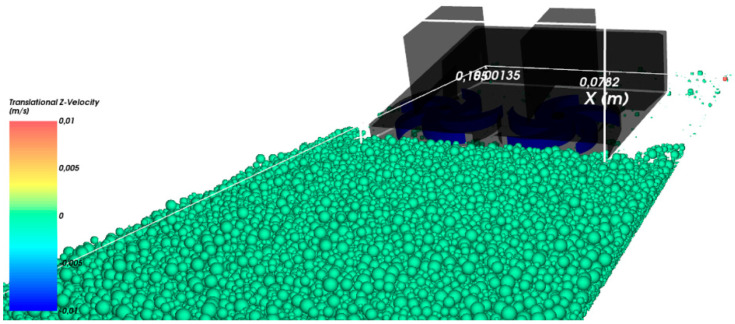
Starting conditions—isometric view with box transparency option.

All geometries used in the simulations were prepared in the .stl format and the initial conditions for each simulation were the same as it is shown in Figure 3 and Figure 4. The filling of the container with particles took place by sprinkling and free arrangement of the particles under the influence of gravity—the goal was to achieve the desired bed height—the same as the top cutting edge and equal to 12 mm.

In the presented simulations, spherical particles were used with the sieve size varying from 0.5 to 4 mm. The DEM solver used the model based on the hysteric linear spring formula for the calculation of normal forces and the linear spring coulomb limit model for the tangential forces. To determine the rolling resistance, the linear spring rolling limit model was used. The static and dynamic friction coefficient between particles was defined as 0.7 and between machine parts and particles as 0.3. The values used in the calculations are the ones that are most often used by scientists in similar simulations [20,21].

**Figure 5:**
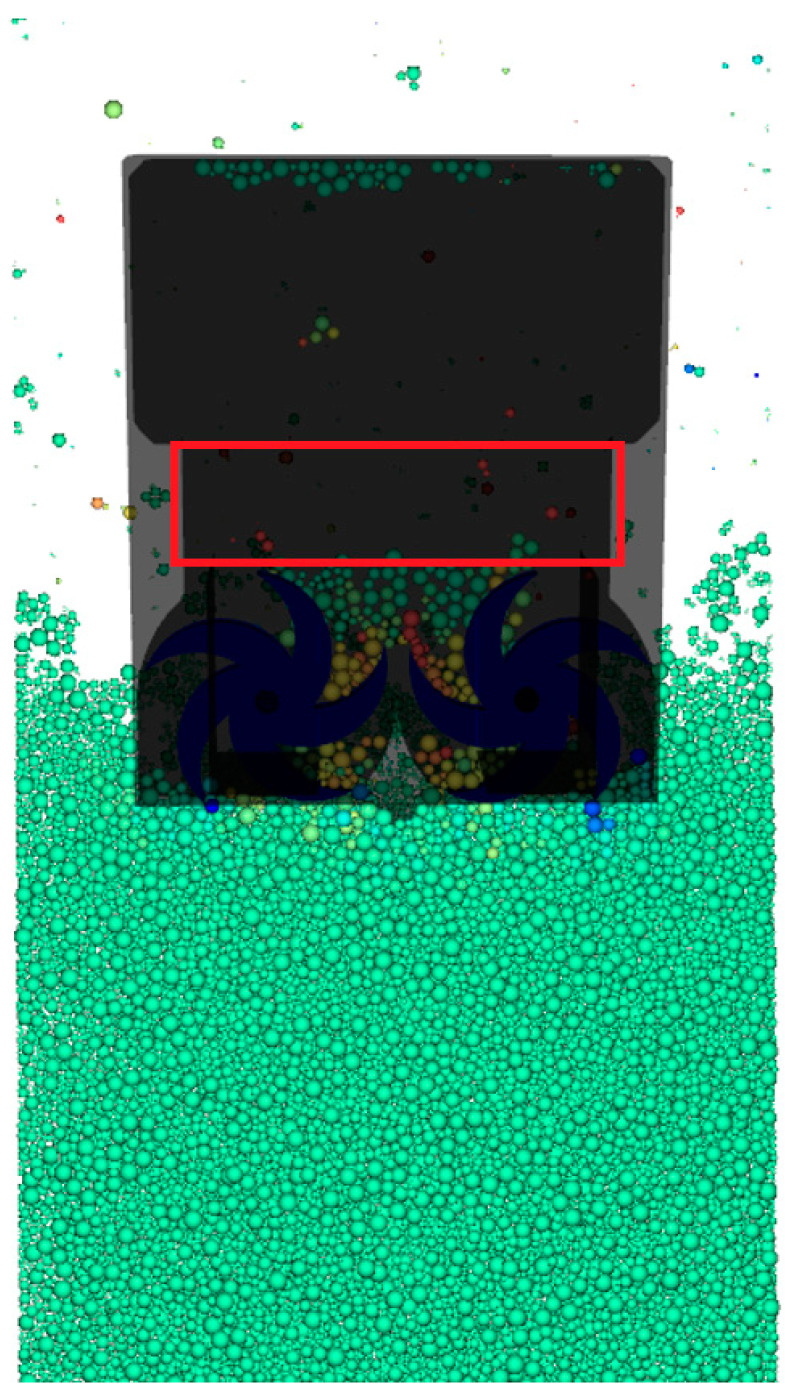
Particle collection process using DEM modeling. The color of the particles indicates their linear velocity. Red box presents the place where the collected particles are calculated.

Total tangential stiffness in contact was also assumed. Such assumptions are best suited to ideal spherical shapes used in the experiment. Due to limited computational capabilities, the duration of the simulation was limited to 8 s. During this time, the harvester moved at a speed of 0.01 m/s, and its blades rotated at a speed of 2 rad/s, collecting spherical particles. Despite such a short simulation time, computing on one GPU card took over 24 h for each calculation. In Section 5, the results obtained for three different values of gravitational acceleration, corresponding to those on the Earth, Moon, and Mars, are presented. Collecting process is shown in Figure 5.

To verify the correctness of the model calculations, experimental tests were carried out and compared with the simulation results for the earth gravity value.

## 4. Experimental Test Stand and Harvester Prototype

The vast majority of the test bench elements and the entire prototype were made with the use of 3D printing techniques. To prepare the 3D CAD models, the SolidWorks software was used.

### 4.1. Harvester Prototype

The first technology used to make the drive elements, blades, display housing, and the first version of the harvester’s body was FDM/FFF printing. FDM It is the simplest and cheapest technology that allows for the creation of relatively large elements (in relation to SLA and SLS). The method is based on fusing the filament at a temperature of ~483 K and the material is applied layer by layer. The material obtains its final strength properties by cooling in the air. Parts that have been properly designed in the CAD program do not require any special processing after being collected from the printer—they are ready to be assembled. The advantages of this technology are:

- The low cost of the printer;
- The low operating costs (material costs ~17 $/kg);
- The large printing space (up to 300 mm × 300 mm × 400 mm).

**Figure 6:**
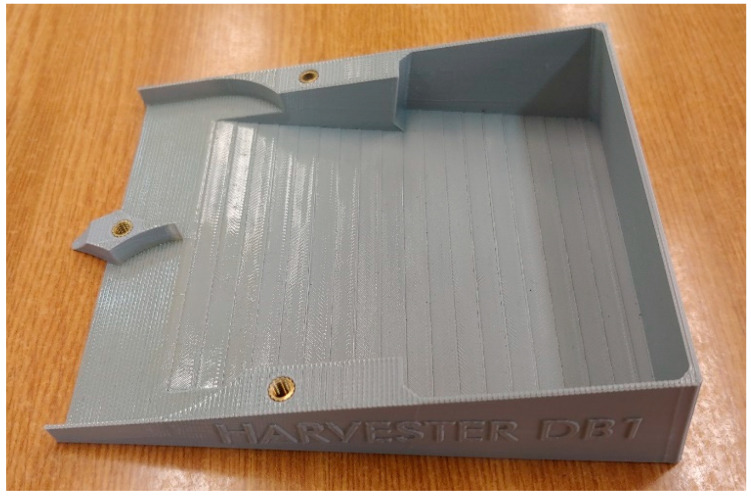
Harvester main body produced using the FDM method.

The main disadvantage of this technology is the relatively low accuracy of the part (compared to other 3D printing technologies). In the X/Y plane, the accuracy is on the level of 0.2 mm and in the Z direction, depending on the layer settings, it is in the range of 0.1 mm to 0.3 mm. The main part of the machine manufactured with this technology is shown in Figure 6.

Initial tests showed that the accuracy of the FDM printout was not sufficient for this element. Particles during the test got stuck on the steps created during printing. Therefore, SLA technology was used to create the second version of the harvester’s body.

**Figure 7:**
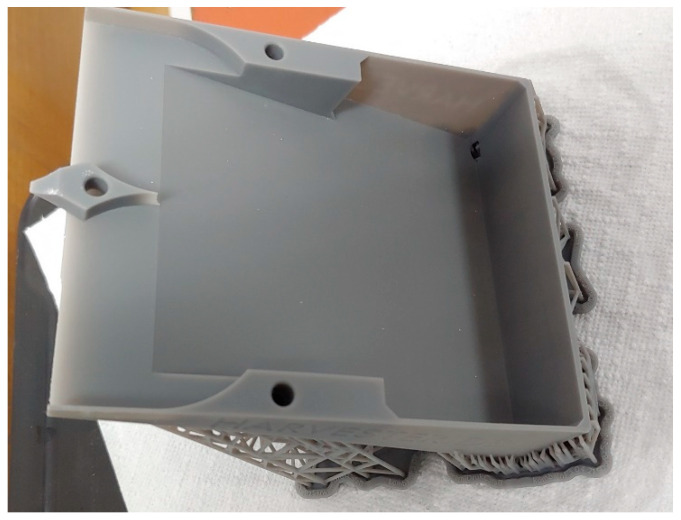
Harvester main body produced using the SLA method.

The SLA technology consists of hardening a liquid photopolymer with a UV laser beam. The object, as in any 3D printing technology, is created layer by layer, while this technology achieves a much higher resolution and, thus, higher accuracy of the produced parts. The main advantage of the SLA technology is that it has the highest accuracy/resolution among 3D printing technologies. For Formlabs FORM 3 printer, it is 0.025 mm in the XY plane and 0.025–0.300 mm in the Z direction (depending on the layer set). This printer uses the LFS (low-force stereolithography) technology, which reduces the forces tearing the part when removing individual print layers from the FEP film. As a result, the printed elements have an exceptionally smooth surface and are free from imperfections. This technology is much more expensive than FDM, a liter of photopolymer costs from $180 to $340. Photopolymer resin is toxic and can cause chemical burns, therefore, it is necessary to use appropriate protective equipment and a dedicated workplace. Parts leaving the printer are not ready for use and require further processing. The first step is to wash off any remaining uncured resin with an isopropyl alcohol bath. After bathing in IPA, the printout requires 3 h of “standing” until the remnants of the solution evaporate from the element. This process can be accelerated by heating the part to 323 K. The next step is the final hardening of the parts with UV light. The last stage of processing is removing the supports, which are always present in this technology, and grinding the joints. A harvester base produced using SLA technology is shown in the Figure 7.

**Figure 8:**
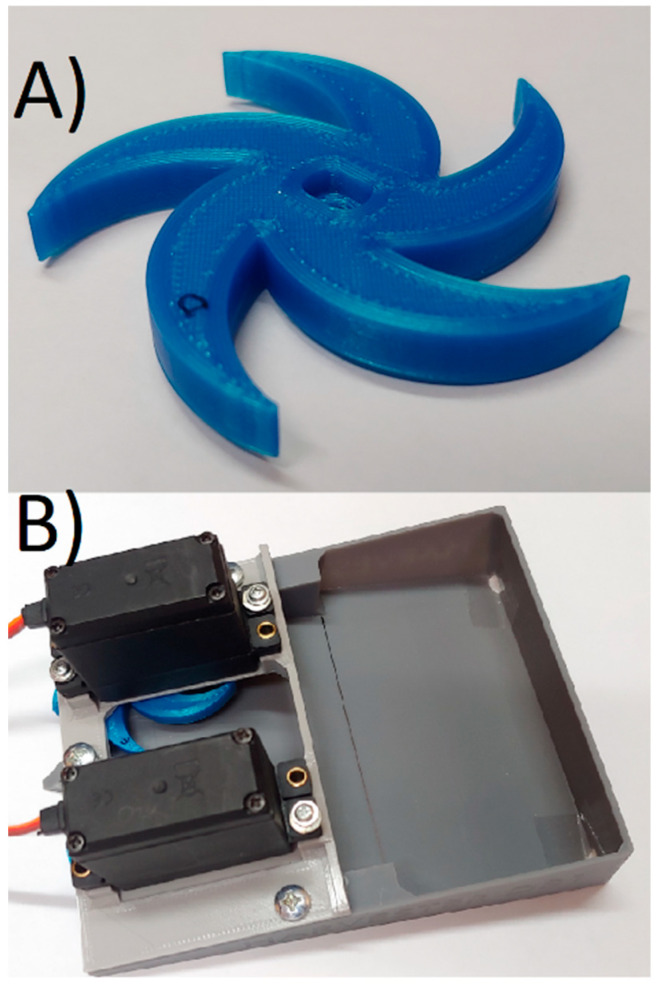
(**A**) 3D printed blade, (**B**) fully assembled harvester.

The rotating blades of the machine were made using the FDM technique for both bodies. The blade and the entire assembled machine are shown in Figure 8.

All the main tests presented in this article were performed using a harvester main part printed with the SLA technology.

### 4.2. Test Stand

**Figure 9:**
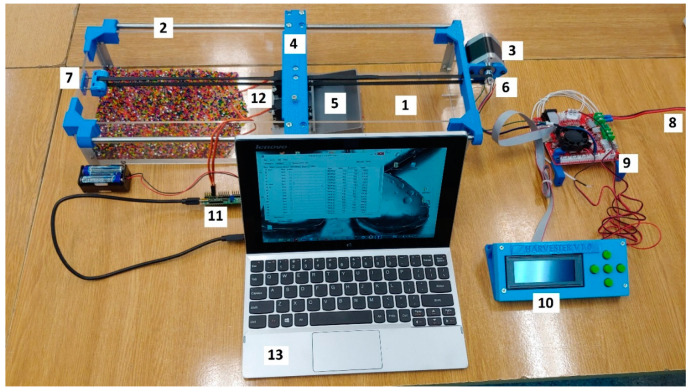
Test stand with equipment: 1—plexiglass box, 2—linear guide, 3—stepper motor, 4—supporting beam, 5—harvester, 6—belt transmission, 7—adjustable tensioner, 8—power supply, 9—microcontroller, 10—control box with display, 11—servo controller, 12—servo motors, 13—PC.

The test stand, presented in the Figure 9, was designed to allow easy adjustment of the angle of attack and the height of the harvester above the material. For this purpose, brass M4 inserts were installed in the bodywork. They were hot stamped in the FDM printout. In the SLA printout, the tolerances of the insert holes had to be changed because the hardened resin does not melt under the influence of heat, but degrades. In this technology, the inserts were mounted in larger holes with glue.

The tests were carried out inside a box made of transparent plexiglass (1). On the top of the box were mounted linear guides (2) and a stepper motor (3). The harvester (5) was mounted to the supporting beam (4), which was moving on the guides driven by a stepper motor using a belt transmission (6). An adjustable tensioner (7) was installed on the belt transmission to avoid slippage. The harvester consisted of a body, top cover, and two high-torque servo motors (12). Two independent control systems were used to control the system. The first was responsible for controlling the stepper motor—the harvester feed. It consisted of a 12 V power supply (8) and a SKR 1.3 driver (9) equipped with a processor, to which a stepper motor and a display (10) were connected. The previously written GCODE program saved on the SD card was started using the display screen. The second system was responsible for controlling the servo motors. It consisted of a servo Maestro 24-Channel USB Servo Controller (11) powered by a 6V battery, to which two servos were connected. The control was carried out using a laptop (13) equipped with Maestro Control Center software and connected via USB to the controller. Two independently controlled servos were intentionally used to avoid blocking the system in case one of the blades met resistance. In this configuration, the second blade would continue to spin at the set speed even with the first blade completely locked.

## 5. Results

This chapter presents the results related to two issues: an attempt to determine the correlation between the simulation result and the conducted experiment and an attempt to compare the results obtained in the simulation with different gravitational conditions. The main limitations of the proposed method are also described.

### 5.1. Experimental Investigations Results

**Figure 10:**
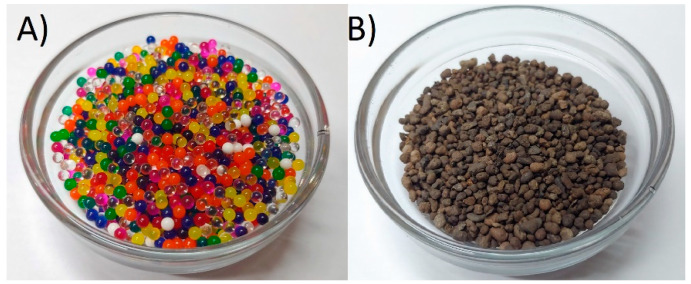
Particles used in the investigations: (**A**) polymer balls, (**B**) expanded clay.

Two different particles’ material and shape were used in the investigations, expanded clay as a substitute of a material with complex shapes and polymer balls as an idealized example of particles, similar to that used in simulations. Both particles are presented in the Figure 10.

In order to obtain the appropriate diameters in the expanded clay substrate, the material was sieved through a sieve with a mesh of 3 mm so that the maximum size of the element was not larger in any direction than the spheres used in the simulation. This material is characterized by non-spherical particles with high surface roughness and, thus, with smaller bulk density than polymer balls.

**Figure 11:**
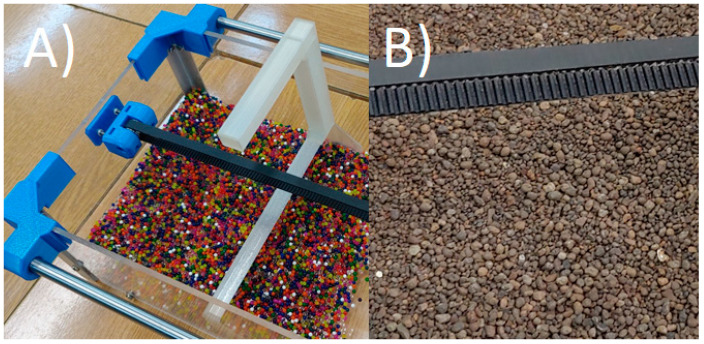
Test beds: (**A**) polymer particles during distribution, (**B**) expanded clay distributed soil.

All experimental investigations were performed using a predefined algorithm that was developed to bring the experimental conditions as close as possible to the simulation conditions. The procedure consisted of five steps:

1. Particles were poured into the box, which were distributed using a specially prepared tool (Figure 11) to obtain the appropriate material bed height.
2. The harvester was set in the correct position, just before the particle bed.
3. The servo motors were run and the rotation speed set at 2 rad/s.
4. Camera record run with the slow motion option (240 frames/s).
5. Harvester feed started with a speed of 0.01 m/s.

**Figure 12:**
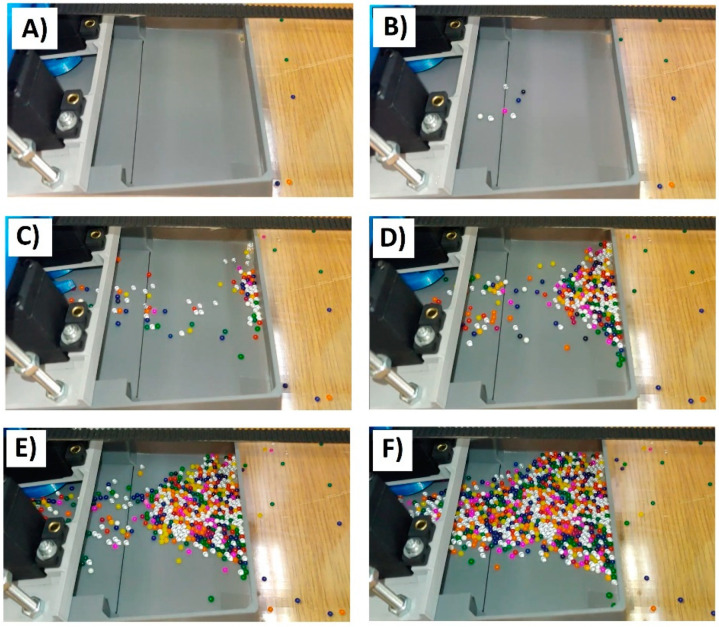
Freeze frames used to calculate the number of particles collected by the harvester at different time points (which passed the black dividing line): (**A**) experiment start (0 s), (**B**) after 1.5 s, (**C**) after 3 s, (**D**) after 4.5 s, (**E**) after 6 s, (**F**) after 7.5 s.

The whole process was repeated ten times for each case and the obtained results were averaged. Unfortunately, in many cases the material was jammed in the blades and the blades became immobilized. Investigations performed using FDM-produced harvester were unsuccessful. For both materials, the blades was jamming and tests were interrupted. The situation was much better when the SLA-produced body was used. However, the non-uniform expanded clay shapes still generated so much trouble that not a single 8 s experiment could be carried out without the blade jamming. Only by idealizing the geometries so that they corresponded to the geometry from the DEM model, i.e., using polymer balls with a diameter of 1–4 mm and a smooth body, was it possible to conduct a full 8 s test. The behavior of the system in this case was also very similar to that of the simulation. The most interesting similarity was the characteristic bursts of balls appearing due to the short-term concentration of stresses between particles and rotating blades. After the tests, slow-motion video was analyzed in order to count the balls loaded into the machine at 0.5 s intervals. An example of the freeze frames are shown in Figure 12.

**Figure 13:**
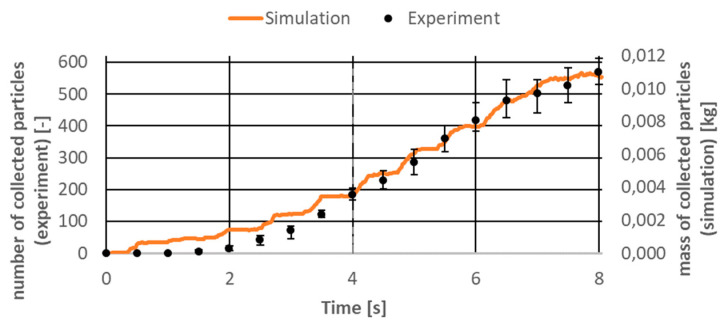
Simulation and experimental results comparison.

Successful experimental studies were compared with the results obtained using the DEM model. A model was used for the comparison in which the boundary conditions corresponded to the terrestrial conditions. Figure 13 shows a comparison of the particle collection rate for the simulation time of 8 s. As the simulation was developed and took into account particles of different sizes in the 0.5–4 mm range, two different parameters were compared, being their analogs and allowing for a qualitative analysis of the results. The number of particles collected by the machine in the experiment was compared to the mass of collected particles in the DEM simulation.

**Figure 14:**
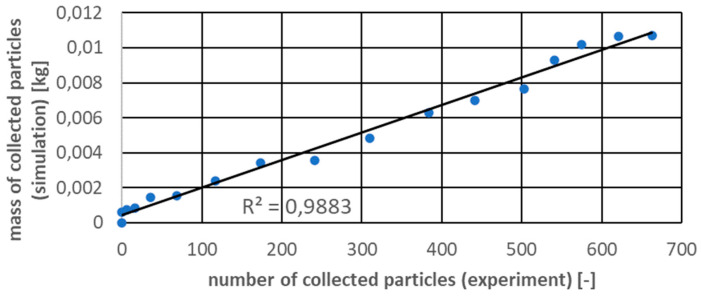
Correlation between chosen parameters.

As it can be seen in the Figure 13, the simulation results were in good agreement with the experimental results for the selected quantities. Although it is difficult to notice it on the graph, up to 4 s the differences between the average value measured in the experimental tests and the deviations were the largest and even reached 40%. This proves that the first seconds of interaction between the machine and the bed of particles are highly unpredictable. After 4 s, the differences were already around 8% and, as can be seen, the DEM results were within the uncertainty ranges in this qualitative analysis. In the Figure 14, the correlations between these parameters for different time points are presented.

Investigations showed very good agreement between the simulation and experiment with the Pearson correlation coefficient equal to 0.9883. In this case authors decided to use the described DEM model to compare the influence of gravity on the regolith harvesting.

### 5.2. Simulation Results

The presented DEM model, positively verified on the test stand, was used to perform comparative calculations of machine–particle interactions under various gravitational conditions. Apart from the conditions corresponding to the terrestrial conditions, calculations were made for the gravitational values on the moon and mars. The adopted values of gravitational acceleration are presented in Table 1.

**Figure 15:**
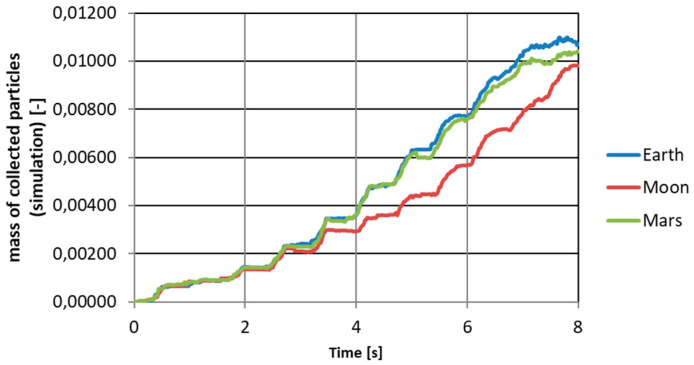
Mass of collected particles in time for different gravitational conditions.

For such defined conditions, leaving the remaining boundary conditions as those described in Section 3, the intuitively expected results were obtained—the lower the gravity value, the weaker the effect of collecting particles and the greater the effect of scattering them in the air. In Figure 15, the masses of collected particles vs. time for three different gravity conditions are presented.

**Figure 16:**
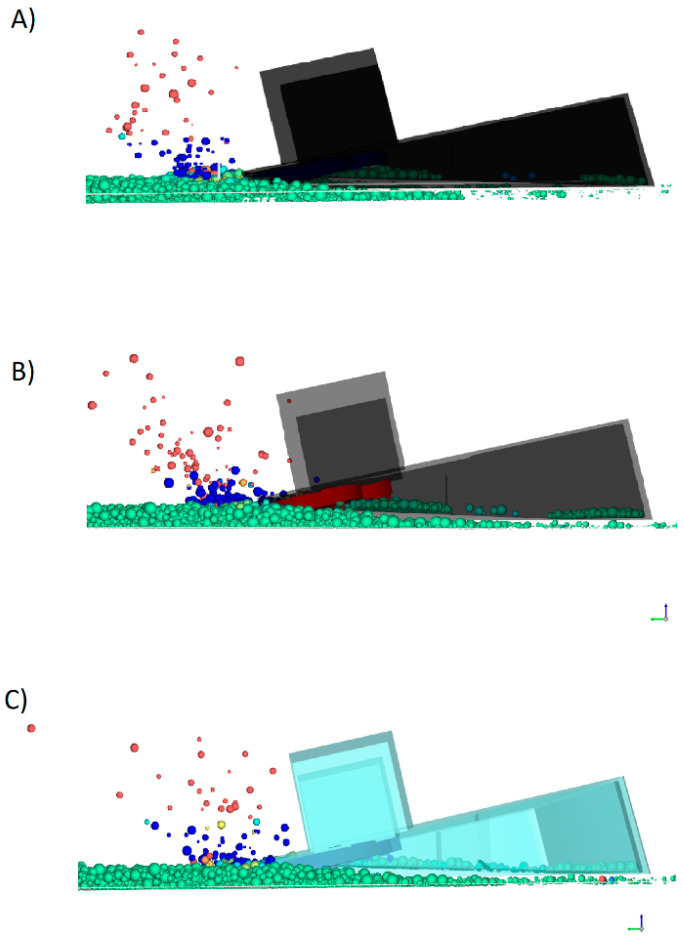
Particle “shots” for: (**A**) Earth gravity, (**B**) Mars gravity, (**C**) Moon gravity values.

One of the decisive factors influencing the results is certainly the effect of gravity on the shots of particles above the machine body and the surface layer of the collected material. Figure 16 shows the highest and widest particle shots for all three cases.

It is hard to visualize it on the picture, but for lower values of gravity, the particles stayed longer in the air and flew away from the harvester at greater distances than for higher values of gravity. Table 2 shows a comparison of the number of particles lifted in the machine–particle interaction during an 8 s simulation.

**Figure 17:**
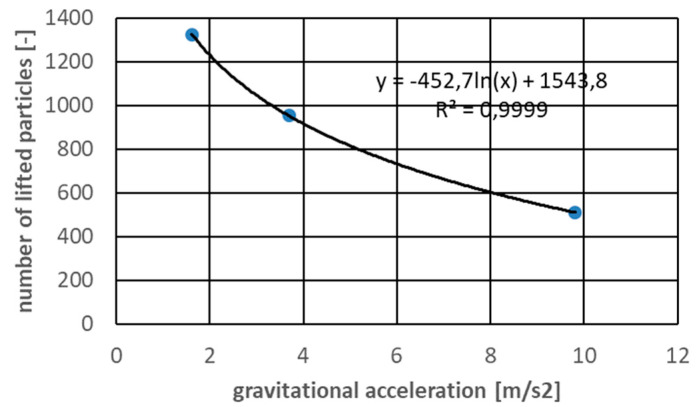
Number of lifted particles in different gravity conditions.

The presented simulation results suggest that the gravity drop becomes important only when is very high. Differences between accelerations of 9.81 m/s^2^ and 3.70 m/s^2^ were not as significant as might be expected. In Figure 17, the logarithmic correlation between lifted particles and gravity with a high Pearson correlation factor is presented. The most important question related to this correlation that should be asked is whether it is the effect of the physics of the phenomenon or is it the effect of a defined numerical model? This should certainly be noted in the future.

The results obtained using the prepared DEM model show that it is possible to use this type of simulation for the qualitative assessment of the interaction between the machine and soil particles under various gravitational conditions. However, the approach presented is a very simple model that can only be used for a preliminary assessment.

## 6. Conclusions

As presented in the article, it is possible to effectively model, under various gravitational conditions, simple phenomena concerning the interaction of soil and machine. Of course, more accurate models are necessary, but this article was intended to show how quickly and easily you can develop devices at an early, conceptual stage. The article also briefly describes what problems may arise when idealized shapes are used in the simulation and the experimental model is always characterized by a certain imperfection. While it is not a problem to generate particles with a variety of randomly generated shapes, when preparing machine elements in the CAD software, designers always aim for the most perfect shape. In order not to complicate the process of creating 3D models, one option is the very dynamic development of 3D printing techniques, which in many cases already allow for obtaining perfect shapes. There is still a need to develop and refine these types of models in order to accurately determine the behavior under different gravitational conditions, but it is a great hope and an opportunity for the future.

## Figures and Tables

**Table 1:** Gravitational conditions used in DEM simulations.

Case	Gravity acc [m/s^2^]
Earth	9.81
Mars	3.70
Moon	1.62

**Table 2:** Particles lifted during simulation.

Case	Number of Particles	Increase
Earth	508	0
Mars	956	88.2%
Moon	1323	160.4%
